# Studies on the Identification of the SND Gene Family in *Pinus massoniana* Lamb. and the Function of *PmSND4* in Secondary Cell Wall Formation

**DOI:** 10.3390/plants15111684

**Published:** 2026-05-29

**Authors:** Yidan Song, Qianzi Li, Sheng Yao, Xuan Lou, Laiwang Sun, Yuchuan Hu, Kongshu Ji

**Affiliations:** 1 State Key Laboratory of Tree Genetics and Breeding, Nanjing Forestry University, Nanjing 210037, China; syd@njfu.edu.cn (Y.S.); qianzili@njfu.edu.cn (Q.L.); yaosheng0817@njfu.edu.cn (S.Y.); louxuan321@163.com (X.L.); 17516758856@njfu.edu.cn (L.S.); 18751825766@njfu.edu.cn (Y.H.); 2 Co-Innovation Center for Sustainable Forestry in Southern China, Nanjing Forestry University, Nanjing 210037, China

**Keywords:** *Pinus massoniana*, SND gene family, *PmSND4*, secondary wall, wood formation

## Abstract

SND (Secondary wall-associated NAC domain) transcription factors are core regulators of secondary cell wall formation in plants. To identify key genes involved in wood formation in *Pinus massoniana* Lamb. and elucidate the molecular regulatory mechanisms underlying its wood formation, this study systematically characterized the SND gene family in *P. massoniana* and performed functional validation of the candidate gene *PmSND4*. Candidate sequences were obtained by screening the transcriptome database, and SND family members were identified by combining Pfam domain validation. Bioinformatics analysis was used to analyze their phylogenetic relationships and conserved motif characteristics. Tissue expression profiling was employed to screen potential candidate genes, and the molecular characteristics of *PmSND4* were analyzed through subcellular localization. Poplar was used as a heterologous expression host to validate its biological function. The results showed that six *P. massoniana* SND family members (*PmSND1-6*) with complete NAC domains were identified. *PmSND4* belongs to the SND/ANAC075 subfamily, and is preferentially expressed in stems, with relatively lower and comparable expression levels in needles and roots. It is localized in the nucleus. Overexpression of *PmSND4* significantly promoted the vegetative growth of transgenic poplar, increased stem xylem cell wall thickness, and significantly enhanced lignin and hemicellulose contents, but had no significant effect on cellulose content. This study completed the systematic identification of the SND gene family in *P. massoniana* and clarified the effect of *PmSND4* on lignin and hemicellulose accumulation in transgenic poplar, providing important gene resources and a theoretical basis for understanding its potential role in wood quality improvement in *P. massoniana*.

## 1. Introduction

*Pinus massoniana* Lamb. is a unique fast-growing coniferous species native to tropical and subtropical regions of southern China. It is characterized by strong adaptability, wide distribution, and diverse uses, serving as an important tree species for both timber and rosin production, as well as a pioneer species for afforestation in barren mountains [1,2]. As a premier timber species, *P. massoniana* plays a crucial role in forestry production due to its substantial stem biomass and excellent wood basic density [3]. Furthermore, its wood is characterized by a high cellulose content (typically 40–50% of the dry weight) and long tracheid fibers, making it an ideal raw material for the pulp and paper industry. Rosin processed from its resin is also a major forestry export product of China [4,5]. Furthermore, *P. massoniana* forests provide significant ecological benefits in carbon sequestration, climate regulation, water conservation, and windbreak and sand fixation [6]. Therefore, improving the yield and quality of *P. massoniana* wood and cultivating new varieties with superior wood properties hold substantial economic and ecological value.

However, due to the long growth cycle and complex genetic background of *P. massoniana*, traditional breeding methods struggle to meet the urgent demand for superior wood properties in the short term [7,8]. Leveraging modern molecular biology techniques to delve into the molecular regulatory mechanisms of wood formation has become a crucial approach to advancing the genetic improvement of *P. massoniana* wood properties [9]. Wood formation is a continuous developmental process involving cambial cell proliferation, differentiation and expansion of xylem cells, deposition of secondary cell wall (SCW) materials, and programmed cell death [10,11]. The SCW is a thickened layer deposited inside the primary wall, primarily composed of lignin, cellulose, and hemicellulose. These three components intercross to form the basic framework of the cell wall, providing plants with mechanical support, water transport capabilities, and resistance to biotic and abiotic stresses [12,13,14].

Lignin is a phenylpropanoid polymer, categorized into three main types based on its monomeric composition: syringyl lignin (S-lignin), guaiacyl lignin (G-lignin), and p-hydroxyphenyl lignin (H-lignin) [15,16]. Gymnosperm lignin primarily consists of G-units with small amounts of H-units, whereas angiosperm lignin is mainly composed of S-units [17]. Lignin biosynthesis is an intricate process initiated via the shikimic acid pathway, followed by the phenylpropanoid pathway generating cinnamic acid and its derivatives, and finally the specific metabolic pathway reducing these derivatives to lignin monomers [18,19]. Key enzymes include L-phenylalanine ammonia-lyase (PAL), cinnamate-4-hydroxylase (C4H), 4-coumarate: CoA ligase (4CL), cinnamyl alcohol dehydrogenase (CAD), and cinnamoyl-CoA reductase (CCR). Changes in the expression of these enzymes directly affect lignin content and composition, consequently impacting plant mechanical strength and stress tolerance [20,21,22].

Cellulose is a linear polysaccharide composed of β-1,4-linked D-glucose units, constituting 40–90% of the SCW [23]. Its biosynthesis is catalyzed by the cellulose synthase complex (CSC), a rosette-shaped structure assembled from multiple cellulose synthase A (*CesA*) subunits [24,25]. In *Arabidopsis thaliana*, *CesA4*, *CesA7*, and *CesA8* are specifically involved in SCW cellulose synthesis, and mutations in these genes lead to xylem collapse and abnormal growth and development [26,27]. Hemicellulose is a heterogeneous polysaccharide polymer, including xylan, xyloglucan, mannan, and β-(1,3;1,4)-glucan. In gymnosperms, mannan is the primary hemicellulose component, whereas xylan dominates in angiosperms [28,29]. Hemicellulose connects with cellulose via hydrogen bonds and with lignin through ester linkages between its glucuronic acid carboxyl groups and lignin benzyl alcohols, playing a crucial role in maintaining cell wall structural integrity [30,31]. Several genes involved in hemicellulose synthesis have been identified, such as *IRX9*, *IRX10*, and *IRX14* in *Arabidopsis*, whose mutations lead to thinner SCWs and reduced stem mechanical strength [32,33].

SCW biosynthesis is precisely controlled by a complex regulatory network composed of various transcription factors [34,35]. Among these, the SND subfamily (Secondary wall-associated NAC domain) of NAC transcription factors acts as the top-level “master switch,” playing a central regulatory role in SCW formation [36,37]. The SND subfamily corresponds to the ANAC075 clade in the *Arabidopsis* NAC family classification, so named after its founding member *ANAC075*. Its members are collectively referred to as SND/ANAC075 throughout this study. SND subfamily members activate a cascade of downstream transcription factors and SCW biosynthetic structural genes by binding to the secondary wall NAC binding element (SNBE) in the promoters of target genes [38,39]. In *Arabidopsis*, *SND1* is specifically expressed in xylem fibers; its overexpression induces ectopic SCW deposition, while its suppression thins fiber cell walls [40,41]. *VND1–VND7* play key roles in vessel cell differentiation [42,43]. In woody plants like poplar and eucalyptus, SND homologs such as *PtrWNDs* and *EgWND1* have also been confirmed to participate in regulating wood formation [10,44]. Furthermore, ANAC075 is shown to be an upstream positive regulator of VASCULAR-RELATED NAC-DOMAIN7 (VND7) and, possibly, its close homologues, NAC SECONDARY CELL WALL THICKENING PROMOTING FACTOR1 (NST1), NST2 and NST3/SECONDARY WALL-ASSOCIATED NAC DOMAIN PROTEIN1 (SND1), which encode the main regulators of secondary cell wall formation [45]. Given this, genes of the SND/ANAC075 subfamily can be considered as possible key regulators of wood formation in pine.

Although the identification and functional analysis of the SND gene family in angiosperms are relatively advanced [46,47], significant evolutionary differences exist in the molecular mechanisms of wood formation between gymnosperms (conifers) and angiosperms. Systematic identification and functional characterization of the SND gene family in *P. massoniana* have not been reported. Systematic identification of gene families combined with tissue expression profiling is an effective approach to rapidly pinpoint candidate genes functioning in target tissues. In this study, using *P. massoniana* as material, we comprehensively identified the SND gene family, analyzed its phylogenetic characteristics, gene structure, and tissue expression patterns, and screened out *PmSND4* as a promising candidate gene associated with wood formation. Subsequently, we validated its function through subcellular localization and heterologous expression in poplar, focusing on its differential effects on SCW components. This study aims to enrich the molecular theory of wood formation in conifers and provide key targets for molecular breeding of *P. massoniana*.

## 2. Results

### 2.1. Systematic Identification and Bioinformatics Analysis

#### 2.1.1. Phylogenetic Analysis

To investigate the phylogenetic relationships among the SND gene family members in *P. massoniana*, the six identified PmSND protein sequences were aligned with NAC transcription factor family members from *Arabidopsis*, and a phylogenetic tree was constructed using the maximum likelihood method.

**Figure 1:**
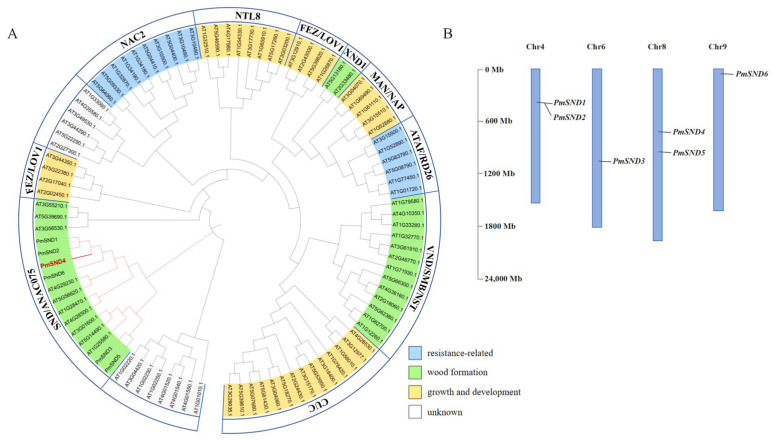
Phylogenetic analysis of the *Pinus massoniana* SND gene family. (**A**) Phylogenetic tree constructed using the maximum likelihood method based on amino acid sequences. Bootstrap values were calculated from 1000 replicates. Red branches indicate *PmSND* family members, with *PmSND4* highlighted in bold red font. (**B**) Chromosomal locations of *PmSND1-6* on *P. massoniana* chromosomes. The scale on the left represents chromosome length (Mb). Chromosome numbers are indicated at the top. Black segments mark the positions of each gene, with gene names labeled on the right.

The results showed that *PmSND1-6* from *P. massoniana* clustered into the same clade as the SND subgroup members in *A. thaliana*, indicating that *PmSND1-6* from *P. massoniana* also belongs to the SND/ANAC075 subgroup. The target gene, *PmSND4*, clustered closely with the *Arabidopsis* SND/ANAC075 subfamily members *AtSND2* (AT4G28500), *AtSND3* (AT1G28470), *AtSND4* (AT5G56620), and *AtSND5* (AT4G29230) within the same branch (Figure 1A). Members of this subfamily are widely demonstrated in model plants to participate in regulating secondary cell wall formation [36,40]. In conclusion, the phylogenetic analysis clarified that *PmSND4* belongs to the SND/ANAC075 subfamily, suggesting its potential involvement in regulating secondary cell wall formation in *P. massoniana*, providing an evolutionary basis for subsequent functional studies. Chromosomal localization analysis showed that *PmSND1-6* were unevenly distributed on chromosomes 4, 6, 8, and 9. Specifically, *PmSND4* and *PmSND5* were both located on chromosome 8; *PmSND1* and *PmSND2* on chromosome 4; *PmSND3* on chromosome 6; and *PmSND6* on chromosome 9 (Figure 1B). This distribution pattern indicates that *P. massoniana* SND family members are relatively dispersed in the genome, suggesting that the family may have primarily expanded through single-gene duplication events. Notably, *PmSND1* and *PmSND2* are located in close physical proximity on chromosome 4, suggesting that these two genes may have originated from a tandem duplication event and could exhibit functional redundancy. To avoid potential challenges in distinguishing individual gene functions during phenotypic characterization, we prioritized the chromosomally independent *PmSND4* as a promising candidate gene for further investigation.

#### 2.1.2. Conserved Motif Analysis of the *P. massoniana* SND Gene Family

**Figure 2:**
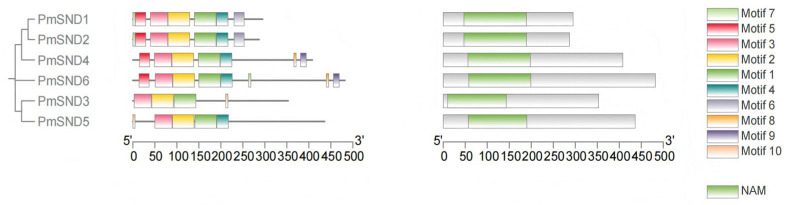
Conserved motif analysis of the PmSND protein family. The left side shows a phylogenetic tree constructed based on conserved motif composition. The right side displays the distribution of conserved motifs (Motif 1–10) for each member. Different colored boxes represent different conserved motifs, with box length indicating the relative position of the motif within the protein sequence. The green box on the right of each PmSND protein indicates the position of the NAM domain. The scale at the bottom represents protein sequence length (amino acids).

To investigate the protein structural characteristics of the *P. massoniana* SND gene family members, the MEME online tool was used to predict conserved motifs in the amino acid sequences of *PmSND1-6*. A total of 10 conserved motifs (Motif 1–10) were identified, and a phylogenetic relationship was constructed based on motif composition (Figure 2).

The conserved motif analysis revealed that all *PmSND* family members contained Motif 1, Motif 2, and Motif 3. These three motifs are core components of the NAC domain, confirming that these members belong to the NAC transcription factor family. The target gene *PmSND4* contained Motifs 1–5, 8, and 9. Notably, Motif 4, which is widely present in SND/ANAC075 members and considered characteristic of this subfamily [36], was also identified in *PmSND4*, further validating its classification within the SND/ANAC075 subfamily. The motif compositions of other family members showed some variation: *PmSND1* and *PmSND2* both contained Motif 1–7; *PmSND3* contained Motif 1–3 and Motif 10; *PmSND5* contained Motif 1–4 and Motif 10; *PmSND6* contained Motif 1–5 and Motifs 7–9. The differences in motif composition among members suggest potential functional diversification, consistent with their distribution across different branches in the phylogenetic analysis. In conclusion, the conserved motif analysis confirmed that *PmSND4* possesses the typical motif features of the SND/ANAC075 subfamily, providing a structural basis for its potential role in regulating secondary cell wall formation.

### 2.2. Tissue Expression Profiling of the SND Gene Family and Screening of PmSND4

To investigate the tissue-specific expression patterns of *P. massoniana* SND gene family members, real-time quantitative PCR (RT-qPCR) was employed to analyze the relative expression levels of the six *PmSND* genes in roots, stems, and needles. *PmTUA* was used as the reference gene, and expression levels were normalized to the root tissue (set as 1.0).

**Figure 3:**
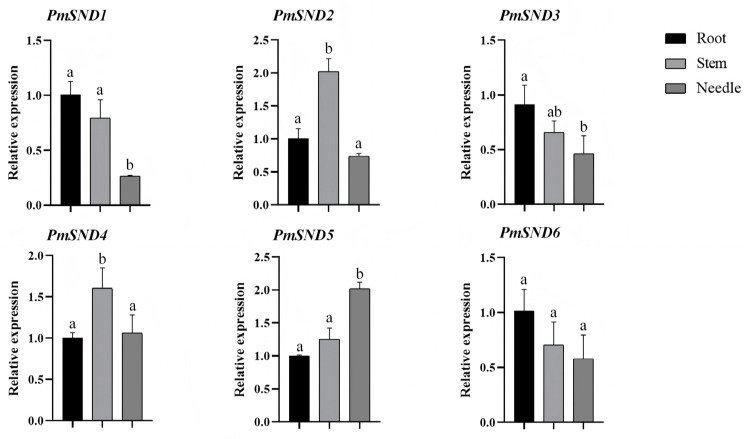
Tissue expression profiles of *PmSND* genes in different tissues. Relative expression levels of six *PmSND* genes in roots, stems, and needles were analyzed by real-time quantitative PCR. *PmTUA* was used as the reference gene, and expression levels were normalized to the root tissue (set as 1.0). Black, light gray, and dark gray bars represent root, stem, and needle tissues, respectively. The vertical axis represents relative expression level, and the horizontal axis represents gene names. Data are presented as mean ± SD (*n* = 3). Different lowercase letters indicate significant differences at *p* < 0.05 (one-way ANOVA, Duncan’s multiple range test).

The expression profiling results (Figure 3) demonstrated that *P. massoniana* SND gene family members exhibited significant tissue-specific expression patterns, suggesting functional diversification. Specifically, *PmSND1*, *PmSND3*, and *PmSND6* displayed their highest expression levels in roots. This root-dominant expression pattern indicates that these members may play specific regulatory roles in root development or the differentiation of below-ground vascular tissues. Conversely, *PmSND5* showed relatively high expression in needles, suggesting its potential involvement in the formation of photosynthetic tissues and their associated vascular structures or secondary cell wall deposition.

Most notably, both *PmSND2* and *PmSND4* were significantly highly expressed in stems. Given that the stem is the primary organ for wood formation in *P. massoniana*, this stem-preferential expression strongly implies that these two members are critical candidate genes for regulating xylem development and secondary cell wall biosynthesis during conifer wood formation. Among them, *PmSND4* showed relatively high expression levels in stems, while its expression levels in needles and roots were relatively lower and showed no significant difference. This expression pattern aligns with the functional characteristics of SND subfamily members involved in the development of secondary growth tissues. Combined with the phylogenetic analysis results, *PmSND4* was identified as a promising candidate gene associated with wood formation in *P. massoniana* and was consequently selected for subsequent functional validation.

### 2.3. Subcellular Localization of PmSND4

**Figure 4:**
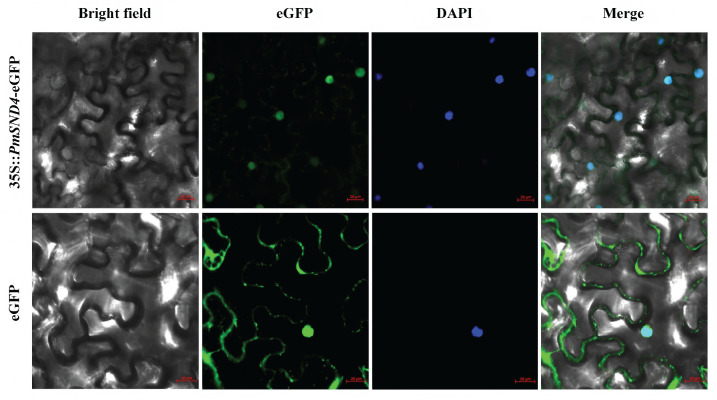
Subcellular localization of *PmSND4* in *Nicotiana benthamiana* leaf epidermal cells. The 35S::*PmSND4*-eGFP fusion vector was transiently transformed into *N. benthamiana* leaf epidermal cells, with the 35S::eGFP empty vector as a control. Fluorescence signals were observed using a laser confocal microscope 48 h post-transformation. Bright field shows cell morphology. Green fluorescence (eGFP) indicates protein localization. Blue fluorescence (DAPI) indicates nuclei. Merge images show the overlay of signals from different channels. Scale bar = 20 μm.

To elucidate the spatial distribution and potential regulatory site of the PmSND4 protein within the cell, a transient expression assay was performed in *Nicotiana benthamiana* leaf epidermal cells. Confocal laser scanning microscopy (Figure 4) revealed that the green fluorescent signal from the 35S::eGFP empty vector (control) was ubiquitously distributed throughout the nucleus, cytoplasm, and cell membrane. In stark contrast, the fluorescence signal from the 35S::*PmSND4*-eGFP fusion protein was exclusively restricted to the nucleus, showing perfect colocalization with the DAPI-stained nuclear regions. These results demonstrate that PmSND4 is a nuclear-localized protein, a characteristic consistent with its predicted role as a transcription factor facilitating the regulation of downstream target genes.

### 2.4. Generation and Phenotypic Analysis of PmSND4 Overexpressing Poplar

**Figure 5:**
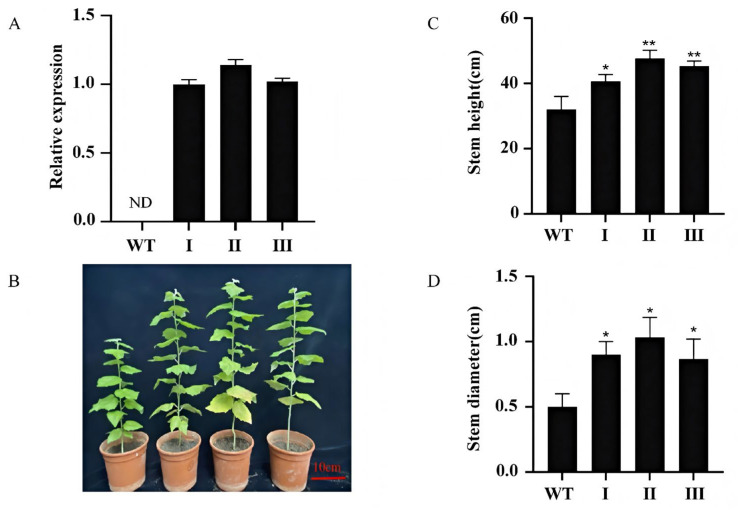
Molecular identification and phenotypic analysis of *PmSND4* overexpressing poplar. (**A**) Relative expression levels of *PmSND4* in wild-type (WT) and three independent transgenic lines (I, II, III) detected by qPCR. Poplar 18S rRNA was used as the reference gene, and expression was normalized to transgenic line I (set as 1.0). ND indicates that the expression of *PmSND4* was not detected in wild-type (WT) plants. (**B**) Representative photographs of 90-day-old wild-type and transgenic plants. Scale bar = 10 cm. (**C**) Statistical analysis of stem height in wild-type and transgenic plants. (**D**) Statistical analysis of stem diameter at the ninth internode in wild-type and transgenic plants. Data are presented as mean ± SD (*n* = 3). Asterisks indicate significant differences between the transgenic lines and the WT (* *p* < 0.05, ** *p* < 0.01, Student’s *t*-test).

Due to the lack of a mature genetic transformation system for *P. massoniana*, poplar was selected as a heterologous expression host for functional validation of *PmSND4* based on codon usage preference analysis (Table S1). The pCAMBIA1301-*PmSND4*-O overexpression vector was transformed into poplar using the Agrobacterium-mediated leaf disc method, and resistant plants were obtained through hygromycin screening. qPCR analysis confirmed the successful expression of *PmSND4* in three independent transgenic lines (I, II, III), with no specific amplification detected in the wild-type (WT). Among them, the expression levels showed no significant difference across the three transgenic lines (Figure 5A).

To investigate the effect of *PmSND4* overexpression on plant growth, the three transgenic lines and the WT control were subjected to phenotypic observation after 90 days of growth. The results showed that the overall growth vigor of the transgenic plants was significantly superior to that of the WT. Statistical analysis of stem height and the diameter of the ninth internode demonstrated that both parameters were significantly higher in the three overexpression lines compared to the WT (*p* < 0.05) (Figure 5B–D). These results indicate that *PmSND4* overexpression significantly promotes vegetative growth in poplar.

### 2.5. Effect of PmSND4 Overexpression on Xylem Anatomy and Cell Wall Composition in Poplar

To investigate the impact of *PmSND4* overexpression on wood formation, the xylem anatomy of transgenic poplar stems was observed, and the main components of the secondary cell wall were quantified.

**Figure 6:**
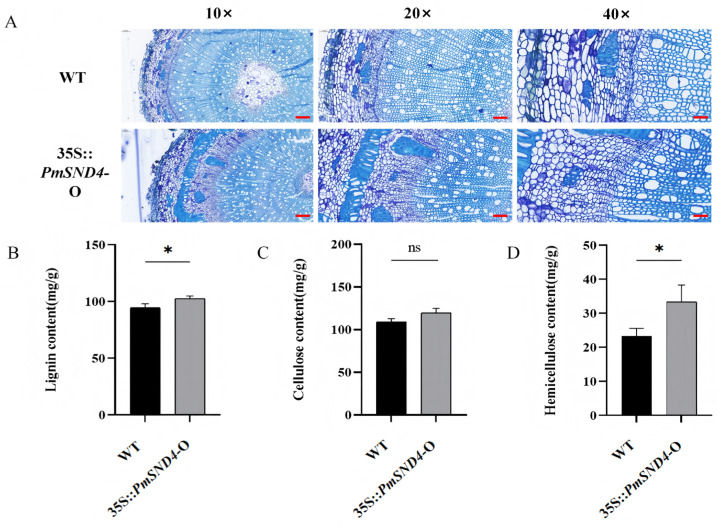
Effects of *PmSND4* overexpression on xylem anatomy and cell wall composition in poplar. (**A**) Toluidine blue-stained cross-sections of stems from wild-type (WT) and 35S::*PmSND4*-O transgenic plants. Images were taken at 10×, 20×, and 40× magnifications; Red bars in each image indicate the scale bars, representing 100 μm, 50 μm, and 20 μm, respectively. (**B**) Lignin content determination. (**C**) Cellulose content determination. (**D**) Hemicellulose content determination. Data are presented as mean ± SD (*n* = 3). Asterisks indicate significant differences between transgenic plants and WT (* *p* < 0.05, Student’s *t*-test). “ns” indicates no significant difference.

Anatomical observation of stem cross-sections revealed that, compared to the wild type, the xylem thickness was significantly increased in the stems of *PmSND4*-O overexpressing transgenic poplar, and the proportion of xylem area within the stem cross-section was markedly higher (Figure 6A). Determination of secondary cell wall components showed that, compared to the wild type, the lignin and hemicellulose contents in the transgenic plants overexpressing *PmSND4* were significantly increased (*p* < 0.05). However, there was no significant difference in cellulose content between the transgenic plants and the wild type (*p* > 0.05) (Figure 6B–D). These results indicate that *PmSND4* overexpression in transgenic poplar primarily promotes the accumulation of lignin and hemicellulose in the secondary cell wall, while showing no significant effect on cellulose content under the tested conditions.

## 3. Discussion

This study represents the first systematic identification of the SND gene family in *P. massoniana*, identifying six members with complete NAC domains. Phylogenetic analysis placed *PmSND4* within the SND/ANAC075 subfamily, whose members are widely recognized as key regulators of secondary cell wall formation in angiosperms [36,40]. Gene structure and conserved motif analyses revealed that *PmSND4* possesses typical characteristics of the SND/ANAC075 subfamily, providing a structural basis for its potential function in regulating secondary cell wall deposition. The dispersed chromosomal localization *P. massoniana* SND family members suggest that their evolution primarily involved single-gene duplication events, which aligns with the evolutionary characteristics of gene families in gymnosperms [47]. These findings are consistent with a recent transcriptome analysis in *Pinus densiflora*, where NAC transcription factors homologous to the SND/ANAC075 subfamily were identified and shown to be preferentially expressed in developing xylem, further supporting the conserved role of this subfamily in secondary cell wall formation across *Pinus* species [48]. Although *PmSND2* also showed high expression in stems, we prioritized *PmSND4* for in-depth functional study due to considerations of genomic context and experimental clarity. The close physical proximity of *PmSND1* and *PmSND2* on chromosome 4 suggests a high risk of functional redundancy, which could complicate the interpretation of phenotypic data. By selecting *PmSND4*, which resides at an independent genomic locus, we aimed to ensure a more definitive and unambiguous characterization of its regulatory role in secondary cell wall formation. However, we acknowledge that this prioritization is primarily a methodological strategy. Biologically, the high stem expression of *PmSND2* suggests it may play an equally critical role in *P. massoniana* wood formation. Therefore, our current findings do not imply that *PmSND4* is uniquely important or functionally superior to *PmSND2*. Rather, *PmSND4* serves as an effective representative member of this subfamily for our initial functional validation in regulating secondary cell wall deposition.

Tissue expression profiling revealed that *P. massoniana* SND gene family members exhibited divergent tissue-specific expression patterns. Notably, *PmSND2* and *PmSND4* were preferentially expressed in stems, consistent with the functional characteristics of SND genes regulating secondary cell wall formation. The high expression of *PmSND4* in stems makes it an ideal candidate gene for regulating wood formation in *P. massoniana*, and its tissue-specific expression suggests that its function may be mainly concentrated in organs undergoing secondary growth.

Nuclear localization is a fundamental prerequisite for transcription factors to bind target gene promoters and exert transcriptional regulation. Our subcellular localization assay confirmed that *PmSND4* is exclusively targeted to the nucleus, which is entirely consistent with its predicted role as a transcriptional regulator. Having established its correct spatial positioning within the cell, we next sought to investigate its biological function in vivo.

Due to the absence of a mature genetic transformation system for *P. massoniana*, this study employed poplar as a heterologous expression host to validate the function of *PmSND4*. Poplar, as a model woody plant, shares a certain degree of conservation in secondary cell wall formation mechanisms with *P. massoniana* [10], making heterologous expression results informative for reflecting *PmSND4* function. Overexpression of *PmSND4* led to increased xylem thickness and cell wall thickening in poplar, ultimately resulting in significant increases in plant stem height and diameter. This establishes a logical chain from gene expression to cellular structure to whole-plant phenotype, suggesting a potential positive regulatory role of *PmSND4* in secondary cell wall formation when heterologously expressed in poplar.

A notable observation in this heterologous system is the differential effect of *PmSND4* on secondary cell wall components. In angiosperms such as *Arabidopsis* and poplar, members of the SND/ANAC075 subfamily typically coordinately regulate the synthesis of cellulose, lignin, and hemicellulose [10,41]. However, this study found that *PmSND4* significantly promoted only the accumulation of lignin and hemicellulose, with no significant effect on cellulose synthesis when heterologously expressed in poplar. It should be noted that the current observations are based on bulk compositional measurements with a limited sample size, representing conditional effects. Therefore, the absence of a cellulose phenotype in this study does not preclude the possibility that *PmSND4* may affect cellulose biosynthesis at different developmental stages, in other tissue types, or under different growth conditions. Further investigation with extended time points and additional analytical methods is warranted. It remains to be determined whether this observed functional divergence reflects evolutionary differences in the molecular regulatory networks of wood formation between gymnosperms and angiosperms, or if it represents a conditional response specific to the heterologous poplar system. Although this study utilized a heterologous expression system, the high expression of *PmSND4* in *P. massoniana* stems correlates well with the cell wall component changes observed in transgenic poplar, supporting its potential conserved role in secondary cell wall formation.

From an industrial application perspective, the potential of *PmSND4* to influence cell wall composition warrants further investigation. In the pulp and paper industry, cellulose is the core utilized product, while lignin is the main component that needs removal, with high content substantially increasing production costs and environmental burden. The observation that *PmSND4* modulated lignin and hemicellulose contents without significantly altering cellulose levels under our specific heterologous assay conditions makes it a candidate gene of interest for wood quality research [22]. However, considering the conditional nature of these effects, future comprehensive validations in *P. massoniana* across different developmental stages and environmental conditions are essential. If such conditional modulation patterns hold true in its native host, targeted manipulation of *PmSND4* could eventually offer theoretical strategies for cultivating customized timber varieties, such as lower-lignin pulpwood or higher-strength materials.

This study also has certain limitations. Due to the immature genetic transformation system for *P. massoniana*, the function of *PmSND4* could not be verified in the native species. Future work should focus on optimizing the *P. massoniana* genetic transformation system to generate *PmSND4* knockout and overexpression materials for in planta functional validation. Furthermore, the downstream target genes of *PmSND4* remain unidentified. Specifically, without expression analyses (e.g., qPCR) of lignin- and hemicellulose-related genes in the transgenic lines, its direct role as a transcriptional regulator remains inferred. Subsequent studies employing techniques such as ChIP-seq and yeast one-hybrid assays are needed to screen for its direct target genes, clarifying the molecular pathways through which it influences lignin and hemicellulose accumulation. Additionally, the synergistic regulatory relationships among members of the *P. massoniana* SND gene family warrant further investigation, providing new insights for multi-gene cooperative improvement of wood quality in *P. massoniana*.

## 4. Materials and Methods

### 4.1. Plant Materials

Two-year-old *P. massoniana* used for gene expression analysis were obtained from the Masson Pine Seed Orchard of the Xin Yi Forestry Research Institute, Guangdong Province, China. Root, stem, and mature needle tissues were collected, immediately frozen in liquid nitrogen, and stored at −80 °C until use.

Wild-type shoot tip cultures of hybrid poplar (*Populus davidiana* × *Populus bolleana*) used for genetic transformation and *N. benthamiana* plants used for subcellular localization were maintained in our laboratory. Poplar shoot tips were subcultured on Murashige and Skoog (MS) medium supplemented with 0.8 mg/L 6-benzyladenine (6-BA), 30 g/L sucrose, and 7 g/L agar (pH 5.8). *N. benthamiana* seeds were sown in nutrient soil. Both species were grown in a greenhouse under controlled conditions (25 °C, 16 h light/8 h dark photoperiod, light intensity of 2000 lx). Four-week-old *N. benthamiana* plants were used for subcellular localization assays.

### 4.2. SND Gene Family Identification and Bioinformatics Analysis

A local BLASTp search using BLAST+ 2.14.0 (E-value < 1 × 10^−5^) was performed against the *P. massoniana* transcriptome database using the amino acid sequences of *Arabidopsis* SND/ANAC075 subfamily members (*AtSND2/3/4/5*) as queries. The integrity of the NAC domain in candidate sequences was verified using the Pfam database (http://pfam.xfam.org/, accessed on 26 December 2025). Sequences without a complete NAC domain were discarded. The confirmed *P. massoniana* SND family members were named *PmSND1* to *PmSND6*.

A phylogenetic tree was constructed using MEGA-X software (version 10.1.8) with the maximum likelihood method based on amino acid sequences. Bootstrap analysis was performed with 1000 replicates. *Arabidopsis* protein sequences were retrieved from NCBI (see Table S2 for sequences). Gene structure (exon/intron organization) was visualized using the GSDS 2.0 online tool (http://gsds.gao-lab.org/, accessed on 23 January 2026). Conserved motifs were predicted using the MEME online tool (https://meme-suite.org/meme/, version 5.5.0, accessed on 30 January 2026) with the following parameters: maximum number of motifs = 10, motif length = 6–50 amino acids. Chromosomal localization was visualized using TBtools software (version 1.098) based on *P. massoniana* genome annotation information.

### 4.3. Tissue Expression Profile Analysis

Total RNA was extracted from *P. massoniana* roots, stems, and needles using the FastPure Universal Plant Total RNA Isolation Kit (Vazyme Biotech Co., Ltd., Nanjing, China) following the manufacturer’s protocol. RNA integrity was checked by 1% agarose gel electrophoresis, and concentration and purity were determined using a NanoDrop 2000 spectrophotometer (Thermo Scientific, Waltham, MA, USA). RNA samples with OD260/280 ratios between 1.8 and 2.1 were used for first-strand cDNA synthesis with the HiScript III First-Strand cDNA Synthesis Kit (Vazyme), according to the manufacturer’s instructions.

The *P. massoniana* tubulin gene *PmTUA* (GenBank accession No. KM496535.1) was used as an internal reference gene [49]. Gene-specific qPCR primers for SND family members were designed (primer sequences are listed in Table S3). Quantitative real-time PCR (qPCR) was performed using Hieff UNICON^®^ Universal Blue qPCR SYBR Green Master Mix (Yeasen Biotechnology Co., Ltd., Shanghai, China) on an ABI Step One Plus Real-Time PCR System (Applied Biosystems, Foster City, CA, USA). The 10 μL reaction mixture contained: 1 μL of diluted cDNA (corresponding to 50 ng total RNA), 0.4 μL each of forward and reverse primers (10 μmol/L), 5 μL SYBR Green Master Mix, and 3.2 μL ddH_2_O. The thermal cycling program was: 95 °C for 30 s, followed by 40 cycles of 95 °C for 10 s and 60 °C for 30 s. Three biological replicates (samples from three independent plants) and three technical replicates were performed for each sample. Relative expression levels were calculated using the 2^−ΔΔCt^ method [50], normalized to the expression level in root tissue (set as 1.0). Statistical analysis was performed using SPSS 22.0 software (SPSS Inc., Chicago, IL, USA). One-way ANOVA followed by Duncan’s multiple range test was used to determine significant differences (*p* < 0.05). All graphs were generated using GraphPad Prism 9.5.0 software.

### 4.4. Subcellular Localization Analysis

The coding sequence of *PmSND4* without the termination codon was amplified by PCR from *P. massoniana* cDNA. Primers incorporating *BamH* I and *Xba* I restriction sites were designed (primer sequences are listed in Table S3). The purified PCR product and the pJIT166-GFP empty vector were double-digested with *BamH* I and *Xba* I (TaKaRa Bio, Shiga, Japan). The digested fragments were ligated using the ClonExpress II One Step Cloning Kit (Vazyme Biotech Co., Ltd., Nanjing, China) to generate the 35S::*PmSND4*-eGFP fusion expression vector. The verified recombinant plasmid was transformed into *Agrobacterium tumefaciens* strain GV3101.

Positive agrobacteria were cultured in LB liquid medium containing appropriate antibiotics at 28 °C until OD_600_ = 1.0. Cells were harvested by centrifugation at 5000 rpm for 10 min and resuspended in infiltration buffer (10 mmol/L MES, 10 mmol/L MgCl_2_, 200 μmol/L acetosyringone) to OD_600_ = 1.0, followed by incubation at room temperature for 3–4 h. The suspension was infiltrated into the abaxial side of leaves from four-week-old *N. benthamiana* plants using a 1 mL needleless syringe. Infiltrated plants were kept in the dark for 24 h, then transferred to normal light conditions (16/8 h light/dark) for another 24 h. Leaf epidermal cells from the infiltrated area were stained with 10 μg/mL DAPI for 5 min and observed under an LSM710 laser confocal microscope (Carl Zeiss, Oberkochen, Germany) with excitation wavelengths of 488 nm (eGFP) and 405 nm (DAPI). Agrobacteria carrying the empty pJIT166-GFP vector were used as a control.

### 4.5. Generation and Identification of Transgenic Poplar

#### 4.5.1. PmSND4 Codon Optimization and Overexpression Vector Construction

To select an appropriate host for heterologous expression and enhance the translation efficiency of *PmSND4*, we compared the codon usage of *PmSND4* with the genomic codon usage patterns of several model organisms: *Escherichia coli* (Ec), *Saccharomyces cerevisiae* (Sc), *Arabidopsis thaliana* (At), *Nicotiana tabacum* (Nt), and *Populus* (data obtained from the Kazusa Codon Usage Database). Different species exhibit distinct codon usage biases that are closely linked to their specific expressed tRNA pools. Therefore, optimizing the codon composition of a target gene to match that of the host organism is essential to circumvent translational bottlenecks during heterologous expression [51,52]. Our analysis indicated that *Populus* was the most suitable heterologous expression host among them (see Table S1). Consequently, the open reading frame of *PmSND4* was codon-optimized according to the codon usage pattern of the poplar genome. The optimized sequence, named *PmSND4*-O (sequence comparison before and after optimization is shown in Table S4), was synthesized by Sangon Biotech (Shanghai) Co., Ltd., Shanghai, China. The optimized sequence was cloned into the pCAMBIA1301 vector using primers with *Nco* I and *Bgl* II sites (primer sequences are listed in Table S3), generating the pCAMBIA1301-*PmSND4*-O overexpression vector driven by the CaMV 35S promoter. The verified recombinant plasmid was transformed into *A. tumefaciens* strain EHA105.

#### 4.5.2. Genetic Transformation and Plant Regeneration

Poplar genetic transformation was performed using the leaf disc method [53]. Leaf discs (1 cm × 1 cm) prepared from 30-day-old sterile poplar leaves were immersed in the Agrobacterium suspension (OD_600_ = 0.4) for 20 min. After blotting dry on sterile filter paper, the discs were placed adaxial side up on co-cultivation medium (differentiation medium supplemented with 100 μmol/L AS) and cultured in the dark at 25 °C for 2 days. After co-cultivation, the discs were washed three times with sterile water containing 250 mg/L timentin, transferred to light culture medium (differentiation medium + 250 mg/L timentin), and cultured under light at 25 °C for 7 days. Subsequently, the discs were transferred to selection medium (differentiation medium + 250 mg/L timentin + 25 mg/L hygromycin) and subcultured every 7 days until resistant adventitious shoots emerged. Resistant shoots were excised and transferred to shoot elongation medium (elongation medium + 250 mg/L timentin + 25 mg/L hygromycin) for 20–25 days. Elongated shoots were then transferred to rooting medium (1/2 MS + 250 mg/L timentin + 25 mg/L hygromycin) to induce roots. Rooted plantlets were acclimatized and transferred to the greenhouse for further growth.

#### 4.5.3. Molecular Identification of Transgenic Plants

Genomic DNA was extracted from leaves of putative transgenic poplar using the FastPure Plant DNA Isolation Mini Kit (Vazyme). PCR amplification of the hygromycin resistance gene (*hptII*) and the *PmSND4*-O gene was performed using gene-specific primers (Table S3) to identify positive transformants. DNA from wild-type poplar was used as a negative control, and the pCAMBIA1301-*PmSND4*-O plasmid was used as a positive control.

Total RNA was extracted from leaves of transgenic poplar using MagZol™ Reagent (Sangon Biotech) following the manufacturer’s instructions. RNA reverse transcription was performed as described in Section 4.3. qPCR was carried out as described in Section 4.3 using poplar 18S rRNA gene (GenBank accession No. AY652861) as the reference gene [54] and *PmSND4*-O specific primers (Table S3). These primers were designed to specifically amplify the codon-optimized *PmSND4*-O transgene. No specific amplification was detected in wild-type poplar, confirming the absence of endogenous *PmSND4* expression. Relative expression levels were normalized to transgenic line I (set as 1.0). Three independent transgenic lines showing high expression levels were selected for subsequent phenotypic analysis.

### 4.6. Phenotypic Analysis

Ninety-day-old wild-type and transgenic poplar plants with consistent growth were selected, with three biological replicates per line (i.e., three independent vegetative clones propagated from a single transgenic regenerant). Plant height (from the base of the stem to the apical bud) and the stem diameter of the ninth internode (measured with a caliper) were recorded. Representative plants from each line were photographed.

### 4.7. Xylem Anatomical Observation

Stem segments from the eighth internode of 90-day-old plants were cut into small pieces (2–3 mm thick) and immediately fixed in FAA solution (70% ethanol: glacial acetic acid: 37% formaldehyde = 90:5:5, *v*/*v*) for at least 24 h. Samples were dehydrated through a graded ethanol series, embedded in paraffin, and sectioned to a thickness of 8–10 μm. Sections were deparaffinized, rehydrated, and stained with 0.5% Toluidine Blue O solution (Solarbio Science & Technology Co., Ltd., Beijing, China) for 5 min. After rinsing with distilled water, sections were dehydrated through a graded ethanol series and mounted with neutral balsam. Observations and images were captured using an BX53 optical microscope (Olympus Corporation, Tokyo, Japan) at 10×, 20×, and 40× magnifications.

### 4.8. Determination of Cell Wall Component Contents

Stem xylem tissue from 90-day-old plants was collected, dried at 105 °C for 30 min, then at 65 °C to constant weight, and ground into powder that passed through a 40-mesh sieve.

Lignin content was determined using the sulfuric acid method [55]. Briefly, 0.1 g of sample powder was mixed with 10 mL of 1% acetic acid, vortexed, and centrifuged. The supernatant was discarded, and the pellet was washed once with 5 mL of 1% acetic acid. The pellet was then treated with 3.5 mL of ethanol–ether mixture (1:1, *v*/*v*) for 3 min, centrifuged, and the supernatant discarded. This step was repeated three times. The pellet was dried in a boiling water bath, then 72% sulfuric acid was added, stirred, and left at room temperature for 16 h. After adding 10 mL of distilled water and boiling for 5 min, the mixture was cooled. Then, 5 mL of distilled water and 0.5 mL of 10% BaCl_2_ solution were added, mixed, and centrifuged at 3000 rpm for 5 min. The pellet was washed twice with distilled water. Subsequently, 10 mL of 10% sulfuric acid and 0.1 mol/L K_2_Cr_2_O_7_ solution were added, and the mixture was boiled for 15 min with constant stirring. After cooling, the mixture was transferred to a flask, and 5 mL of 20% KI solution and 1 mL of 0.5% starch solution were added. Titration was performed with 0.2 mol/L Na_2_S_2_O_3_ solution. A blank control (10 mL of 10% sulfuric acid and 0.1 mol/L K_2_Cr_2_O_7_ solution without sample) was titrated simultaneously. Lignin content (%) was calculated using the formula: Lignin content (%) = (a − b) × k × 0.048 × 100/n, where k is the concentration of Na_2_S_2_O_3_ (mol/L), a is the volume of Na_2_S_2_O_3_ consumed by the blank titration (mL), b is the volume of Na_2_S_2_O_3_ consumed by the sample titration (mL), and n is the sample mass (g).

Cellulose content was determined using the anthrone-sulfuric acid method [56], and hemicellulose content was determined using the hydrochloric acid hydrolysis method [55]. Three biological replicates and three technical replicates were performed for each sample.

### 4.9. Statistical Analysis

All data are presented as the mean ± standard deviation (SD). Statistical analyses were performed using SPSS 22.0 software (SPSS Inc., Chicago, IL, USA). Comparisons between two groups were made using Student’s t-test. Comparisons among multiple groups were made using one-way ANOVA followed by Duncan’s multiple range test. A value of *p* < 0.05 was considered statistically significant. All graphs were generated using GraphPad Prism 9.5.0 software.

## 5. Conclusions

This study systematically identified the SND gene family in *P. massoniana*, clarifying the phylogenetic characteristics, gene structures, and tissue expression patterns of its six members. Through expression profiling combined with phylogenetic analysis, *PmSND4* was screened as a promising candidate gene associated with wood formation. It was confirmed to belong to the SND/ANAC075 subfamily, localize to the nucleus and be highly expressed in stems. Functional validation using poplar as a heterologous expression host demonstrated that *PmSND4* overexpression significantly promotes vegetative growth, increases xylem cell wall thickness, and promotes the accumulation of lignin and hemicellulose in the secondary cell wall of transgenic poplar, with no significant effect on cellulose accumulation under the tested conditions. It should be noted that while these results highlight its conserved regulatory potential, the endogenous function of *PmSND4* in *P. massoniana* warrants further investigation. This study provides insights into the conditional functional bias of an SND gene in a coniferous species, enriches the molecular theory of wood formation, and provides a key candidate gene and theoretical basis for understanding wood property improvement in *P. massoniana*.

## Data Availability

The original contributions presented in this study are included in the article/Supplementary Materials. Further inquiries can be directed to the corresponding author.

## Supplementary Materials

The following supporting information can be downloaded at: https://www.mdpi.com/article/10.3390/plants15111684/s1, Table S1. Codon usage preference analysis of *PmSND4* and host selection. Table S2. Protein sequences used for phylogenetic analysis of *PmSND* gene family. Table S3. Primers used in this study. Table S4. Sequence comparison of *PmSND4* before and after codon optimization.
